# Supplementary material for: Randomness-induced quantum spin liquid on honeycomb lattice
Source: Sci Rep. 2017 Nov 23;7:16144. doi: 10.1038/s41598-017-16431-0 (PMC5701036; doi:10.1038/s41598-017-16431-0)
Supplement: Supplementary file 1 — Supplementary Information [file 41598_2017_16431_MOESM1_ESM.doc]

**Randomness-induced quantum spin liquid on honeycomb lattice**

Hironori Yamaguchi*, Masataka Okada, Yohei Kono, Shunichiro Kittaka,

Toshiro Sakakibara, Toshiki Okabe, Yoshiki Iwasaki,

and Yuko Hosokoshi

*Author to whom correspondence should be addressed.

E-mail: yamaguchi@p.s.osakafu-u.ac.jp

**Crystal structure and intermolecular exchange interactions**

The crystals contain two regioisomer with different Cl atom positions, labeled A- and B-type, as shown in Fig. S1a. The ratios of the two molecular patterns, which are given by *x* and 1-*x* for the A- and B-type molecules, respectively, were determined via X-ray single crystal structural analysis through optimization of the Cl occupancy. The crystallographic data for Zn(hfac)2(A*_x_*B_1-_*_x_*) obtained at 293 and 23 K are summarized in Table S1. Only slight differences are apparent between the data obtained at *x*=0.64 and 0.79, and no indication of a structural phase transition is observed to temperatures as low as approximately 25 K in both crystals. For the *x*=0.79 data at 293 K, we fixed the Cl occupancy to that evaluated at 25 K. We primarily focused on the structural features related to the verdazyl radical at 25 K to examine the intermolecular interactions in the low-temperature regions. The verdazyl ring (which includes four N atoms), the two upper phenyl rings, and the bottom pyridine ring were labeled as R1, R2, R3, and R4, respectively, as shown in Fig. S1a. The R_1_-R_2_, R_1_-R_3_, and R_1_-R_4_ dihedral angles were approximately 18°, 48°, and 9° for *x* = 0.62, and 19°, 47°, and 10° for *x*=0.79, respectively. The *ab* *initio* molecular orbital (MO) calculation indicated almost identical spin density distribution for the A-type and B-type molecules for both *x*=0.64 and 0.79. Approximately 58 % of the total spin density was present on R1. While R2 and R3 each account for approximately 18 % of the relatively large total spin density, R4 constituted less than 6 % of the total spin density. Note that, as Zn(hfac)_2_ has a low spin density of less than 1 %, it acts as a spacer between the verdazyl radicals, yielding the observed good two-dimensionality of the lattice. Therefore, the intermolecular magnetic interactions arise through the overlapping of the molecular orbitals on the verdazyl radical.

We evaluated the intermolecular magnetic interactions of molecular pairs at 25 K via the *ab initio* MO calculations. Consequently, we found four types of interactions *J*_1_, *J*_2_, *J*_3_, and *J*_4_, as shown in Fig .2b-e. Each molecular pair associated with the interaction had three pair formation patterns, A-A, A-B (B-A), and B-B, with A-B being equivalent to B-A because of the inversion symmetry between molecules. The A-A, A-B (B-A), and B-B pairs have possibilities of *x*^2^ = 0.41 (0.62), 2*x*(1-*x*)= 0.46 (0.33), (1-*x*)^2^ = 0.13 (0.04), respectively, for *x*=0.64 (0.79). The evaluated exchange interactions are summarized in Table S2. Although the absolute values underwent some changes, the signs did not depend on the pair formation. Note that, as the overlaps of the molecular orbitals for *J*_1_ and *J*_3_ are strongly related to the phenyl rings with randomly distributed Cl atoms, their values are highly dependent on the pair formation. On the other hand, the rings related to the overlaps of the molecular orbitals for *J*_2_ and *J*_4_ have no Cl atom and, thus, their absolute values are almost unchanged. The three dominant interactions, i.e., *J*_1_, *J*_2_, and *J*_3_, form the *S* = 1/2 honeycomb lattice in the *ac*-plane, as shown in Fig. S1b. The honeycomb lattices are separated from each other by nonmagnetic Zn(hfac)_2_, as shown in Fig. S1c. The weak *J*_4_ corresponds to one of the diagonal bridges of the honeycomb lattice. Note that this weak, but finite AF *J*_4_ induces frustration in the lattice. We must consider the fact that intermolecular interactions at less than 1.0 K in their absolute values are generally difficult to evaluate using the *ab initio* MO calculation, because of a strong dependence on the calculation method. Considering the formation of the random-singlet state in the actual system, the weak interaction *J*_4_ may be considerably stronger than the determined value.


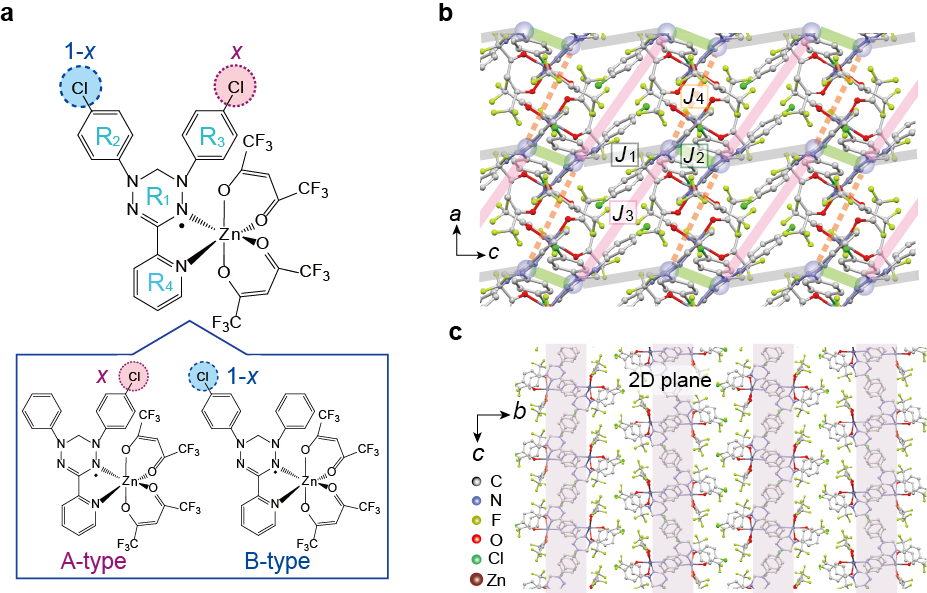


Figure S1: (**a)** Molecular structures of Zn(hfac)2(A*_x_*B_1-_*_x_*). Crystal structure viewed perpendicular (**b**) and parallel (**c**) to the *ac*-plane for *x* = 0.64.

Table S1: Summary of crystallographic data for Zn(hfac)2(A*_x_*B_1-_*_x_*).

| Compound | Zn(hfac)2(A*_x_*B_1-_*_x_*) | | | | | | |
| --- | --- | --- | --- | --- | --- | --- | --- |
| Cl occupancy *x* | 0.639(4) | 0.639(3) | | 0.79 | | 0.786(3) | |
| Temperature (K) | 293(2) | 25(2) | | 293(2) | | 25(2) | |
| Formula | C_29_H_17_ClF_12_N_5_O_4_Zn | | | | | | |
| Crystal system | Monoclinic | | | | | | |
| Space group | *P*2_1_/*c* | | | | | | |
| Wavelength (Å) | 0.7107 | | | | | | |
| *V*(Å^3^) | 3265(3) | | 3107.8(19) | | 3254.9(16) | | 3077.4(19) |
| *a* (Å) | 9.323(4) | | 9.010(3) | | 9.345(3) | | 9.007(3) |
| *b* (Å) | 31.900(15) | | 31.640(11) | | 31.998(9) | | 31.704(11) |
| *c* (Å) | 10.979(5) | | 10.902(4) | | 10.890(3) | | 10.779(4) |
| *β* (degrees) | 90.923(8) | | 90.538(3) | | 91.623(4) | | 91.212(6) |
| *Z* | 4 | | | | | | |
| *D*_calc_(g cm^-3^) | 1.685 | | 1.770 | | 1.690 | | 1.788 |
| Total reflections | 5358 | | 5125 | | 5344 | | 5068 |
| Reflection used | 3632 | | 4598 | | 3949 | | 3647 |
| Parameters refined | 479 | | | | 478 | | 479 |
| *R* [*I* > 2*σ*(*I*)] | 0.0696 | | 0.0436 | | 0.0668 | | 0.0663 |
| *R_w_* [*I* > 2*σ*(*I*)] | 0.1769 | | 0.1065 | | 0.1734 | | 0.1522 |
| Goodness of fit | 1.093 | | 1.033 | | 1.063 | | 1.051 |
| CCDC | 1557999 | | 1558000 | | 1558001 | | 1558002 |

Table S2: Magnetic interactions evaluated through MO calculations at 25 K.

| Pair formation | | A-A | A-B(B-A) | B-B |
| --- | --- | --- | --- | --- |
| *x* = 0.64 | *J*_1_/*k*_B_ | -9.5 K | -14.5 K | -15.8 K |
|  | *J*_2_/*k*_B_ | 7.2 K | 7.1 K | 6.9 K |
|  | *J*_3_/*k*_B_ | 3.9 K | 7.8 K | 10.0 K |
|  | *J*_4_/*k*_B_ | 0.08 K | 0.08 K | 0.08 K |
| *x* = 0.79 | *J*_1_/*k*_B_ | -12.1 K | -16.2 K | -18.3 K |
|  | *J*_2_/*k*_B_ | 6.3 K | 6.1 K | 6.1 K |
|  | *J*_3_/*k*_B_ | 2.1 K | 8.8 K | 13.9 K |
|  | *J*_4_/*k*_B_ | 0.07 K | 0.08 K | 0.09 K |
